# Caregiver distress associated with behavioral and psychological symptoms in mild Alzheimer’s disease

**DOI:** 10.1590/S1980-57642010DN40300013

**Published:** 2010

**Authors:** Ari Pedro Balieiro Jr., Emmanuelle Silva Tavares Sobreira, Marina Ceres Silva Pena, José Humberto Silva-Filho, Francisco de Assis Carvalho do Vale

**Affiliations:** 1 MSc, Psychologist, Behavioral Neurology Group, Clinicas Hospital of the Ribeirão Preto, Faculty of Medicine, University of São Paulo, Ribeirão Preto SP, Brazil.; 2 PhD, Psychologist, Federal University of Amazonas, Psychology Department, Education Faculty, Manaus AM, Brazil.; 3 MD, PhD, Neurologist Faculty of Medicine, Federal University of São Carlos, São Carlos SP, Brazil, and Behavioral Neurology Group, Clinicas Hospital of the Ribeirão Preto Faculty of Medicine, University of São Paulo, Ribeirão Preto SP, Brazil.

**Keywords:** caregiver distress, psychological and behavioral symptoms, Alzheimer’s disease

## Abstract

The aim of this study was to analyze the relationship between Caregiver Distress and Behavioral and Psychological Symptoms in Dementias (BPSD) in mild Alzheimer’s disease.

**Methods:**

Fifty patients and caregivers were interviewed using the Neuropsychiatric Inventory (NPI).

**Results:**

96.0% of the patients had at least one BPSD. The mean NPI total score was 19.6 (SD=18.05; range=0-78) whereas the mean Caregiver Distress Index (CDI) total score was 11.5 (SD=10.41; range=0-40). For the individual symptoms, the weighted mean CDI was 2.8 (SD=1.58). All symptom CDI means were higher than 2.0 except for euphoria/elation (m=1.8; SD=1.49). There were correlations between CDI and derived measures (Frequency, Severity, FxS, and Amplitude) for all symptoms, except Disinhibition and Night-time behavior. Correlations ranged between 0.443 and 0.894, with significance at p<0.05.

**Conclusions:**

All the derived measures, including amplitude, were useful in at least some cases. The data suggests that CDI cannot be inferred from symptom presence or profile. Symptoms should be systematically investigated.

## Abstract (pt)

Analisar as relações entre desgaste do cuidador e Sintomas Comportamentais e Psicológicos nas Demências (SCPD) na doença de Alzheimer leve.

**Métodos:**

Cinquenta pacientes e cuidadores foram entrevistados com o Inventário Neuro-Psiquiátrico (INP).

**Resultados:**

96,0% dos pacientes tinham pelo menos um SCPD. A média do escore total do INP foi 19,6 (dp=18,05; amplitude=0-78). A média do índice de desgaste do cuidador (IDC) foi 11,5 (dp=10,41; amplitude=0-40). Para os sintomas individuais, a média ponderada do IDC foi 2,8 (dp=1,58) (escala 0-5). Exceto para euforia/elação (m=1,8; dp=1,49), as médias dos IDC foram maiores do que 2,0. Foram encontradas correlações entre o IDC e medidas derivadas para todos os sintomas, exceto Desinibição e Sono. As correlações variaram entre 0,443 e 0,894 (p<0,05).

**Conclusões:**

Todas as medidas derivadas foram úteis em algum caso. Os dados sugerem que o IDC não pode ser inferido pela presença ou perfil dos sintomas. Antes, deve ser investigado sistematicamente.

Behavioral and Psychological Symptoms in Dementia (BPSD) is a term used to describe non-cognitive features of dementias since the Consensus group of the International Psychogeriatric Association defined BPSD as “Symptoms of disturbed perception, thought content, mood or behavior that frequently occur in patients with dementia.”^1^ The list of BPSD usually includes personality changes, aberrant behaviors, apathy, agitation, irritability, disinhibition, depression, anxiety, euphoria, dysphoria, delusions, hallucinations and appetite and eating changes. BPSD occur in up to 90% of dementia patients,^2^ and its prevalence in AD is estimated to lie in the 25% to 80% range, depending on the study methodology.^3^ Some studies show that apathy is the most frequent BPSD in patients with AD, while the least common symptom described is euphoria.^4-6^ There is increasingly recognition that BPSD are a different and separate problem to the cognitive decline that characterizes Alzheimer’s disease (AD).^1,7,8^

Furthermore, BPSD are considered an important aspect in the care of dementia patients where investigations have shown that the symptoms are a major source of distress to the family or professional caregivers.^6,9-12^ Symptoms contribute to a reduction in quality of life of caregivers and patients,^13-15^ increase the risk of institutionalization^16,17^ and raise healthcare costs.^18,19^ Studies have shown that BPSD can increase the rate of morbidity in caregivers.^20,21^

Moreover, it has been suggested that caregiver distress is a significant predictor of institutionalization, while behavioral alterations alone are not.^16^ In addition, studies suggest that cultural factors can modify the relationship between BPSD and caregiver distress, although BPSD profiles do not differ significantly.^5,14,22-24^ Another important feature is the early onset of BPSD in the course of AD, which calls for direct attention and demands health, welfare, and medical services.^25^

This study sought to clarify the relationship between BPSD and caregiver distress, by looking for correlations between their different manifestations and caregiver distress, as assessed by the Neuropsychiatric Inventory NPI,^4,25^ a widely recognized instrument for assessing psychopathology in patients with dementia. The study also hypothesizes that the number of different manifestations in each of the domains covered by the NPI is useful to predict caregiver distress, and is proposed as a simple way of obtaining this measure.

## Objectives

The objective of this study was to analyze the correlations between Behavioral and Psychological Symptoms and Caregiver Distress in Mild Alzheimer’s Disease. Correlations were investigated and analyzed separately for each symptom. The presence of correlations between the Caregiver Distress and the symptoms clustered in sub-syndromes was also verified.^29^

## Methods

The study was conducted in a tertiary outpatient clinic (Behavioral Neurology Outpatient Clinic - BNOC - at the Clinicas Hospital of the Ribeirao Preto Faculty of Medicine, University of Sao Paulo). This is a public clinic within an education institution that treats behavioral and cognitive disturbances using a multidisciplinary approach. At present, the casuistic numbers more than 1,500 patients, over half of which have shown dementia syndromes.^32^ This study is part of a larger project investigating BPSD in mild dementias (CDR 0.5 or 1) based on the population assisted by the BNOC.

The inclusion criteria for patients in this sample were: to be a patient at the BNOC registered on the BNOC database; to have been diagnosed with Alzheimer’s disease within the 12 months preceding the interview confirmed by clinical and neuropsychological examination according to the diagnostic criteria of the “*Diagnostic and Statistical Manual, edition IV* (DSM-IV)” and the “*National Institute of Neurologic, Communicative Disorders and Stroke-Alzheimer Disease and Related Disorders Association* (NINCDS-ADRDA)”; and finally to have been rated as CDR 0.5 or 1.0 at interview. The study period spanned from 2004 to 2008. The total number of patients interviewed was 73, although patients rated as CDR greater than 1 at interview were excluded from the final sample.

The inclusion criteria for caregivers in this sample were: being a caregiver accompanying the selected patient during the consultation at the clinic, and who declared to have a reasonable knowledge of the patient, at least enough to answer the questions in the NPI. Caregivers not providing convincing answers to the questions in the interview could be excluded from the sample at the discretion of the interviewer, but all caregivers gave sound answers.

### Demographics

#### Patients

the sample of patients included 50 subjects, 30 women (60.0%), aged 55-94 years (*mean*=74.0; SD=8.0). The estimated mean duration of illness was 38.4 months (SD=20.1; range=12-92). Patients had a mean of 4.9 years of education (SD=4.7; range=0-15). MMSE results ranged between 5 and 28 (*mean*=17.6; SD=5.3), for polymodal.

#### Caregivers

the sample of caregivers included 50 subjects, 45 women (90.0%), aged 27-78 years (*mean*=53.9; SD=13.1). In terms of their relationship with the patient, 34.0% of the caregivers were spouses; 40.0% children and 26.0% had some other kind of relationships. Also in regard to their relationship with patients, 64.0% of caregivers reported living with the patient, against 36.0% that did not. Regarding the percentage of care provided to the patient, 50.0% of caregivers declared to be responsible for more than three quarters (>75%) of the care, 20.0% for 50-75% of care, 14.0% for 25-50%, and 16.0% declared to be responsible for less than a quarter of the care (<25%).

#### Instruments

The Clinical Dementia Rating Scale- CDR^26^ is an instrument for staging dementia based on clinical examination of the patient and collateral sources of information such as caregivers or relatives, and six domains of the patient’s cognition and their impact on the patient’s life. The domains of Memory, Orientation, Judgment & Problem-Solving, Community Affairs, Home and Hobbies and Personal Care are scored as 0 (no impairment), 0.5 (questionable impairment), 1 (mild impairment), 2 (moderate impairment) or 3 (severe impairment). An algorithm developed by Morris^27^ provides an overall CDR classification (0, 0.5, 1, 2, or 3). Given these characteristics, it is possible that patients diagnosed as having Alzheimer’s disease by clinical and neuropsychological means, are rated as CDR 0.5, normally considered to indicate Mild Cognitive Impairment, although is also consistent with mild dementia and cortical pattern such as that found in AD.

The Neuropsychiatric Inventory - NPI^4,25,28^ is an instrument for measuring these symptoms. Besides investigating BPSD, the NPI investigates Caregiver Distress (CDI). The concurrent validity of NPI is good and overall reliability (Cronbach’s a) is 0.88.^25^

The NPI consists of twelve items with related questions specifically designed to investigate anxiety, depression/dysphoria, delusions, hallucinations, agitation/aggression, euphoria/elation, apathy/indifference, disinhibition, irritability/lability, aberrant motor behavior, night-time behavior, and appetite/eating changes. Each item consists of a screening question that investigates the presence of the symptom, followed by 7 to 9 questions that investigate the different manifestations of the symptom, which are asked when the answer to the screening question is “yes”.

For each question answered with a “yes”, the caregiver provides an evaluation of the Frequency - F (scale: 1=occasionally, 2=often, 3=frequently, 4=very frequently), and likewise for Severity – S (scale: 1=mild, 2=moderate, 3=severe) of the symptoms. There is a score for each symptom, a derived measure (FxS) that entails multiplying the Frequency index and the Severity index.

Further, when the informant is the caregiver, the NPI evaluates the caregiver distress for each patient symptom selected. The Caregiver Distress Index (CDI) is rated by the informant on a scale from 0-5 points (0=none, 1=minimal, 2=mild, 3=moderate, 4=severe, 5=very severe or extreme). By summing all the CDI scores for each informant, a global CDI score is obtained for that informant. In the present study however, since the aim of the study was to investigate possible correlations between the CDI and each symptom, the data was analyzed differently, i.e., it was observed whether there were any correlations between the CDI for each symptom and the derived corresponding measures (F, S, and FxS).

Moreover, the total number of “yes” answers to sub questions of each subscale was obtained. Hence, a score was produced for the range of different manifestations of the symptoms investigated by that questionnaire item. This index, called Amplitude - A, aimed to evaluate possible correlations between the given range of manifestations and the CDI. The study hypothesis is that the magnitude of A is useful to help predict caregiver distress for some of the symptoms. The number obtained was not normalized so as to keep this measure very simple. Therefore, simply counting the number of yes answers, as outlined above, was considered a suitable method.

Finally, symptoms were clustered as proposed by Aalten et al.^29^ Thus, there are three sub-syndromes:

[1] mood, which includes depression, apathy, night-time behavior, and appetite/eating change (four items, Cronbach’s α=0.63);
[2] hyperactivity, which includes agitation/aggression, euphoria/elation, irritability/lability, disinhibition, and aberrant motor behavior (five items, α=0.73); and
[3] psychosis, which includes hallucinations and delusions (two items, α=0.72).

Anxiety was not included in any of the items, being taken as a separate factor. In the case of the sub-syndromes, according to recommendations of Aalten et al.^29^, the clinically relevant cut-off point was set at an NPI score ≥3.

### Procedures

This study was approved by the Research Ethics Committee of the Clinicas Hospital of the Ribeirão Preto Faculty of Medicine, University of Sao Paulo, in the 183th ordinary meeting held on 07/05/2004. After having defined the study sample and the instruments, patients and their caregivers were informed about the study objectives and methods, and signed the Term of Informed Consent approved by the Research Ethics Committee.

Patients were then assessed using the BNOC protocol, which includes the CDI and the MMMSE. The caregivers were interviewed using the NPI. The data obtained was converted into percentage (%) or mean (*m*) and standard deviation (SD), depending on the case. For the sub-syndromes, the indices and measures (Amplitude, Frequency, Severity and FxS or NPI sub-syndrome index) were summed to obtain a clustered index or measure of each parameter. Spearman’s Correlation Coefficient (*rho*) was determined among the profile measures, CDI and occurrence of BPSD.

The data was analyzed using SPSS 13.0 for Windows.

## Results

### Caregiver distress and occurrence of BPSD

In the study sample, 96.0% of the patients had at least one BPSD, 88.0% had two or more symptoms, and 76.0% showed at least one symptom with a score ≥4. The total mean NPI score was 19.64 (*SD*=18.05; range=0-78). A total of 88.0% of the patients had a total NPI score ≥4. The mean CDI total score was 11.5 (*SD*=10.41; range=0-40). A total of 74.0% of the caregivers had a total CDI score ≥3. The NPI total score and the CDI total score were correlated at rho=0.85 and significant at the 0.01 level (2-tailed). The CDI total score and the NPI total score were also correlated with the total number of BPSD presented (rho=0.83 for both, significant at the 0.01 level, 2-tailed).

**Figure 1:**
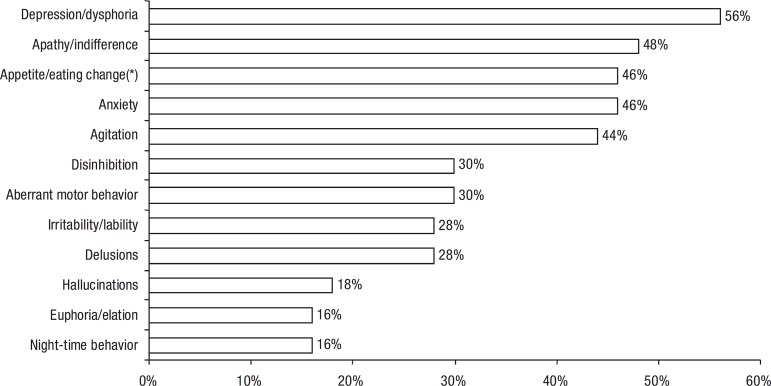
Occurrence of BPSD (n=50).

**Figure 2:**
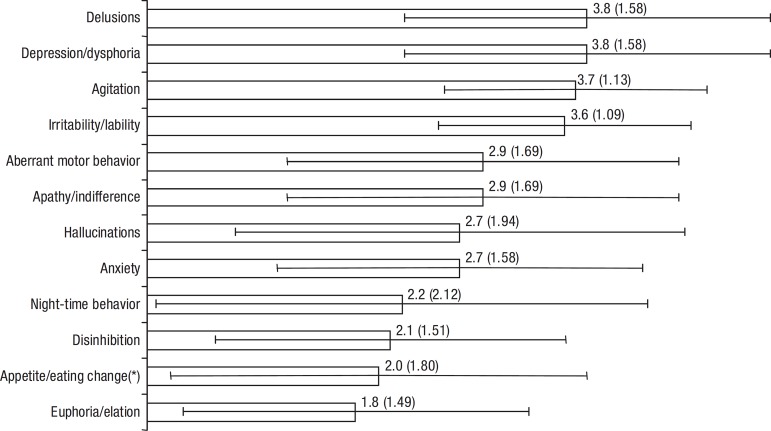
CDI by Symptom - m(SD).

None of the symptoms occurred in more than half of the sample. Only Depression/dysphoria occurred in half of the sample. In contrast, euphoria/elation, hallucinations, and night-time behavior occurred in less than a quarter of the sample. Figure 1 shows the total occurrence of each symptom while Figure 2 shows the Caregiver Distress Index.

### Significant correlations between Caregiver Distress Index and BPSD

Table 1 shows the correlations between the CDI and the BPSD indices found in the data. The table was arranged in descending order of *n* for the occurrence of the symptom. The data shows that all the indices can be correlated with CDI, although not on all BPSD. For example, in the case of Euphoria/Elation, only the FxS index was useful, whereas for Disinhibition and Night-time behavior none of the indices showed any correlation with CDI.

**Table 1:** Significant correlations - caregiver distress × symptom.

Symptom	Amplitude	Frequency	Severity	FxS
Depression/dysphoria (n=28)	-	.696**	.530**	.707**
Apathy/indifference (n=24)	-	.545**	-	.577**
Anxiety (n=23)	-	.443*	.535**	.567**
Appetite/eating change (n=23/49)	-	-	.664**	.558**
Agitation (n=22)	-	-	.425*	-
Aberrant motor behavior (n=15)	.535*	-	.702**	.714**
Disinhibition (n=15)	-	-	-	-
Delusions (n=14)	-	.555*	.782**	.750**
Irritability/lability (n=14)	.792**	-	.894**	.831**
Hallucinations (n=9)	-	-	.847**	.815**
Night-time behavior (n=8)	-	-	-	-
Euphoria/elation (n=8)	-	-	.735*	-
No. of correlations found Modest Strong Total	1 1 2	4 0 4	4 5 9	5 3 8

* Correlation is significant at the 0.05 level (2-tailed);

** Correlation is significant at the 0.01 level (2-tailed).

### Sub-syndromes and CDI

The Mood sub-syndrome was present in 43 patients (86.0%), 30 of whom (69.8%) showed a clinical relevant score (NPI Mood score ≥3). The Hyperactivity sub-syndrome was present in 38 of the patients (76.0%), while 25 of these (65.8%) had an NPI Hyperactivity score ≥3. Finally, the Psychosis sub-syndrome was present in 18 of the patients (36.0%), while 14 of these (77.8%) had an NPI Psychosis score ≥3. Correlations were found, significant at the 0.01 level (2-tailed), between the sub-syndromes and all their clustered measures. Concerning the Mood/apathy sub syndrome, correlations were 0.616 (Amplitude), 0.610 (Frequency), 0.702 (Severity) and 0.654 (FxS or the NPI sub-syndrome score). With regard to the Hyperactivity sub syndrome, the correlations were 0.815 (Amplitude), 0.770 (Frequency), 0.874 (Severity) and 0.793 (FxS). Finally, for the psychosis sub syndrome the correlations were 0.973 (Amplitude), 0.986 (Frequency), 0.998 (Severity) and 0.987 (FxS).

## Discussion

This study examined the BPSD and caregiver distress in a clinic-based sample of patients with Alzheimer’s disease. The merit of this work is the search for correlations between these two features of AD, and clarification of this relationship. Its major weakness is the relatively small sample. The incidence of BPSD in our sample is slightly higher than figures obtained in other studies, which report an incidence of around 80-95%2,^13,31-35^ Another factor to consider is that in this study the majority of caregivers were sons/daughters, while in the cited studies the majority of caregivers were spouses. Nevertheless, Godinho et al.^3^ found no correlation between demographic variables and caregiver distress.

In terms of caregiver distress data, our results differ to those of previous studies with a comparable design.^6^ Further comparisons are difficult since other studies on caregiver distress^14-17,20,21,37,38^ have employed different designs. The strong correlations found between NPI and CDI total scores confirmed the relationship between BPSD and caregiver distress, as previously described in the literature. However, when particular symptoms or details of the correlations are examined, the data shows increased complexity. First of all, it appears that correlations between individual symptoms are not as consistent as those obtained when the symptoms are clustered in some way. However, by observing the symptoms comprising the Hyperactivity sub-syndrome, it was evident that only 8/20(40%) had significant correlations with the CDI, and for the Psychosis sub-syndrome only 3/8(37.5%), and on the Mood sub-syndrome only 5/16(31.3%). Hence, it can be concluded that correlations between clusters exert a statistical effect. This could be useful, although in our view further studies are needed to confirm this issue.

Searching for correlations between all the measures and the partial CDI for each symptom, revealed strong correlations (0.70-0.89) for Amplitude with Irritability; for Severity with Aberrant motor behavior, Delusions, Irritability/lability, Hallucinations and Euphoria/elation; and for FxS with Depression/dysphoria, Aberrant motor behavior, Delusions and Irritability/lability. Modest correlations (0.40-0.69) were detected for Amplitude with Aberrant motor behavior; for Frequency with Depression/dysphoria, Apathy/indifference, Anxiety and Delusions; for Severity with Depression/dysphoria, Anxiety, Appetite/eating change and Agitation; and for FxS with Apathy/indifference, Anxiety and Appetite/eating change.

Despite the relatively small sample, it is believed that these data support the hypothesis that Amplitude can be a useful index, especially because it is very easy to obtain from NPI data. However, Amplitude shows only two significant correlations with CDI. Interestingly, these two symptoms were Aberrant motor behavior (n=15; rho=0.535) and Irritability/lability (n=14; rho=0.792),i.e. productive symptoms, from a psychiatric perspective.

Examination of the magnitude of the correlations revealed that Frequency showed only modest correlations, Amplitude had one strong correlation, FxS three while Severity had five Since Severity contributed to FxS this fact suggests that the severity of the symptoms is probably the major source of caregiver distress.

Finally, since evidence in the literature shows a wide variability of BPSD patterns, the findings of the present study suggest that each symptom, together with the corresponding level of caregiver distress, should be investigated separately.
